# The Ubiquity of Intraguild Predation among Predatory Arthropods

**DOI:** 10.1371/journal.pone.0028061

**Published:** 2011-11-23

**Authors:** Annie-Ève Gagnon, George E. Heimpel, Jacques Brodeur

**Affiliations:** 1 Département de phytologie, Université Laval, Québec, Canada; 2 Department of Entomology, University of Minnesota, St-Paul, Minnesota, United States of America; 3 Institut de recherche en biologie végétale, Département de sciences biologiques, Université de Montréal, Montréal, Canada; Hungarian Academy of Sciences, Hungary

## Abstract

Intraguild predation (IGP) occurs when one predator species attacks another predator species with which it competes for a shared prey species. Despite the apparent omnipresence of intraguild interactions in natural and managed ecosystems, very few studies have quantified rates of IGP in various taxa under field conditions. We used molecular analyses of gut contents to assess the nature and incidence of IGP among four species of coccinellid predators in soybean fields. Over half of the 368 predator individuals collected in soybean contained the DNA of other coccinellid species indicating that IGP was very common at our field site. Furthermore, 13.2% of the sampled individuals contained two and even three other coccinellid species in their gut. The interaction was reciprocal, as each of the four coccinellid species has the capacity to feed on the others. To our knowledge, this study represents the most convincing field evidence of a high prevalence of IGP among predatory arthropods. The finding has important implications for conservation biology and biological control.

## Introduction

Contemporary ecologists struggle with complexity. Communities involve thousands of species interacting in many diverse ways within the spatial and temporal variability of natural ecosystems [1]. In the late 1980's it became apparent that models based on functional trophic levels were not sufficiently universal to understand the dynamics and structure of communities [2]. The necessity of integrating non-trophic and indirect relationships has prompted theoretical and empirical work aimed at examining the role of omnivores. One form of omnivory is intraguild predation (IGP), where one predator species attacks another predator species with which it also competes for a shared prey species [3].

Following the pioneering field study of Polis and McCormick [4] on species of desert scorpions that feed on each other, a fertile and rapidly growing literature on IGP has led to a reconsideration of several classical topics in ecology such as stability and diversity of communities, trophic cascades in food webs, niche shift and species exclusion, as well as the effects of ecosystem productivity on species interactions [3], [5]–[8]. IGP also rapidly became relevant to aspects of applied ecology such as biological control, management of endangered species and the establishment of exotic invasive predators [9]–[11]. IGP is now considered to be ubiquitous in aquatic and terrestrial ecosystems, occurring in a great diversity of taxa from bacteria to mammals [3]. According to an analysis conducted by Arim and Marquet [12] using 113 food webs, 58–87% of animal species are involved in IGP interactions.

Despite this apparent ubiquity of intraguild interactions in both natural and managed ecosystems, and despite the importance of these interactions in structuring communities, very few studies have quantified rates of IGP in various taxa under field conditions. This is especially true for predatory arthropods, most likely because of the perceived difficulty of performing field observations of predation events [13]. Intraguild interactions among arthropod species have traditionally been studied in Petri dishes [14], or in field cage experiments [15]–[18]. Although important for identifying potential functional trophic and guild links among species, these approaches are inadequate for predicting the full complexity of both direct and indirect interactions [13], [19]. Consequently, results from experiments conducted in experimental arenas that have a limited number of interacting species and are conducted for short periods of time have led to skepticism about the actual occurrence and significance of IGP in nature [20].

Some studies have examined IGP in more natural settings using different methodological techniques and are important in complementing the less natural enclosure-based experiments. First, a number of semi-quantitative food-web studies documenting the existence (presence/absence) of trophic linkages between omnivores have shown that predators also include predatory species in their diet [4]. Second, purely observational field studies have quantified predator-predator interactions [21]. Third, experimental studies have been conducted in which the full, natural community of predators and prey were retained, and there was little if any constraint imposed on predator foraging [22]. Finally, a range of biochemical and molecular techniques have been developed to analyze gut contents and assess the diet of predatory arthropods under field conditions [23].

In this study we assess the nature and incidence of IGP among four species of coccinellid predators (Coleoptera: Coccinellidae) in soybean fields under natural conditions. This system has several favourable attributes for the study of IGP. Coccinellids are generalist predators, voracious both during their larval and adult stages. In soybean fields of Québec, Canada, they can be abundant and naturally play a role in aphid control [24]. They show an aggregative response to prey density [25]–[27], thereby increasing encounter rates with conspecific or heterospecific coccinellids. Furthermore, a number of laboratory or exclusion cage experiments have shown that IGP is potentially a common interaction among coccinellids [14], [28] and have identified major ecological determinants of IGP such as relative size of the protagonists, mobility and aggressiveness, feeding specificity and aphid density [14], [29].

A second advantage for using coccinellids as a model system is that we have developed molecular gut-content analyses to assess levels of IGP [30]. This approach uncovers predation events without interfering with the behavior of predators and prey and without disrupting ecosystem processes [31], [32]. Gut-contents analysis using the polymerase chain reaction (PCR) has recently been applied to the study of IGP between predator species [33] and between predators and parasitoids [17], [34].

## Methods

### Ethics statements

No specific permits were required for the described field studies and it did not involve endangered or protected species. Permission to sample invertebrates in the fields was obtained by each grower.

### The study system

We studied the community of coccinellids associated with the soybean aphid, *Aphis glycines* Matsumura (Homoptera: Aphididae), a recent invasive pest in North America [35]. The four dominant species in soybean fields in the province of Québec are: *Coccinella septempunctata* Linnaeus, *Propylea quatuordecimpunctata* Linnaeus, *Harmonia axyridis* (Pallas) and *Coleomegilla maculata lengi* Timberlake, the only native species in this system [36]. These four coccinellid species are sympatric and present throughout the season, with *H. axyridis* arriving later than the others. Their abundance in soybean is mostly correlated with aphid densities, as commonly observed in agroecosystems [37].

Our primary objective was to estimate IGP levels within coccinellid assemblages in soybean fields. For the purposes of this paper, we define the IGP level as the proportion of a sample of a given predator species that contains measurable amounts of DNA of at least one different predator species in their guts. We do not attempt to examine the multitude of ecological factors that can promote the occurrence of IGP (predator and prey densities, predator:prey ratio, predator stage structure, etc) across fields or sampling dates; these analyses will be presented elsewhere. However, to place the present study in context we provide general information about aphid and coccinellid populations. *Aphis glycines* populations were relatively high with seasonal means of 266 and 371 aphids per plant in 2004 and 2005, respectively (A.E. Gagnon, *unpublished data*). The coccinellid community in 2004 was dominated by *H. axyridis* and *C. septempunctata* (representing 48 % and 41 %, respectively, of all four species) with a small proportion of *C. maculata* (5 %) and *P. quatuordecimpunctata* (6 %). In 2005, the proportions of each species were as followed: *H. axyridis* (59 %), *C. septempunctata* (18 %), *C. maculata* (14 %) and *P. quatuordecimpunctata* (9 %).

**Figure 1:**
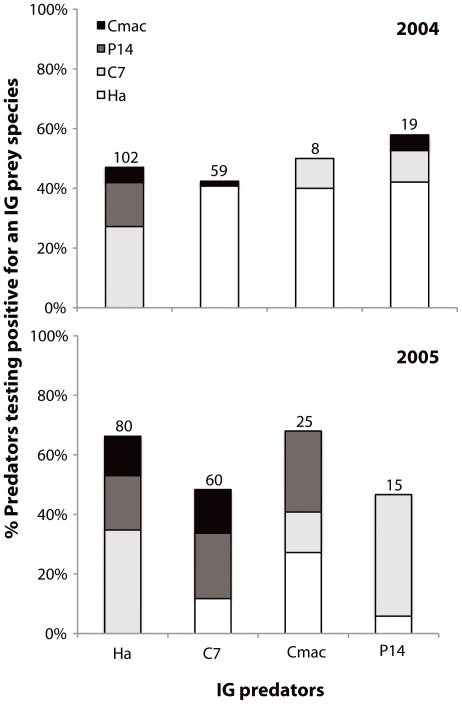
Levels of intraguild predation among four species of coccinellids measured by molecular gut content analysis in soybean fields in Québec, Canada, in 2004 and 2005. Results are expressed as the proportion of each species of intraguild prey detected in the gut of intraguild predators. Ha  =  *Harmonia axyridis*, C7 * =  Coccinella septempunctata*, Cmac * =  Coleomegilla maculata*, P14  =  *Propylea quatuordecimpunctata*. Numbers above histogram bars represent the number of individuals tested.

Coccinellids were sampled in soybean fields in 2004 and 2005 with sweep netting, put in an electric icebox at 4°C, and brought to the laboratory. Specimens were frozen (−20°C) and then washed in 70% ethanol to prevent possible contamination stemming from the time that predators had been held together in the collecting bag [38], [39]. In experiments done by Greenstone *et al.* [40], vigorous beating of plants followed by aspiration of insects into a common dry beaker led to incorrect assignment of gut contents – presumably due to regurgitant or feces from non-prey species contaminating the integument of predators. Contamination in our case is expected to be much lower because insects were immediately chilled rather than aspirated into a common beaker [38], [39]. Also, contamination in the Greenstone *et al.* study was likely particularly high because the prey species they used (larvae of the Colorado potato beetle, *Leptotinarsa decemlineata*) is known to regurgitate readily and in large amounts, and is often covered with secretions and feces that may be particularly prone to generate contamination [40]. Finally, a substantial fraction of the control animals in the Greenstone *et al*. experiment showed contamination, which brings into question the validity of the study (as the authors themselves noted). Samples were preserved in vials with 70% ethanol at 4°C until DNA extraction. Coccinellids were sampled in four different fields, located within the municipalities of Maskinongé (46°12′39″, -73°02′02″), Hérouxville (46°39′59″, -72°37′27″), Nicolet-Sud (46°12′04″, -72°36′47″) and Saint-Augustin-de-Desmaures (46°44′19″, -71°28′43″) in the province of Québec. A total of 188 and 180 coccinellid individuals were sampled in 2004 and 2005, respectively (Figure 1 provides details per species). Insects were sampled from mid-July to mid-September. We only used fourth larval instars in our analyses because they are more likely to be engaged in IGP than are other stages [28].

### DNA extraction and PCR cycles

DNA extraction and PCR protocols were modified from Hoogendoorn and Heimpel [41]. DNA was extracted from whole coccinellid larvae. Each insect was ground in a 1.5 ml microcentrifuge tube using sterile plastic pestles (Ultident Scientific inc.) with 100 µl of grinding buffer [42]. PCR amplifications were done separately for each primer pair (*H. axyridis*; *C. septempunctata*; *C. maculata*; *P. quatuordecimpunctata*). Details of development and cross-reactivity tests of PCR markers are presented in Gagnon *et al.* [30]. All predators were screened against the primers of all three potential intraguild prey and against a universal primer (12Sai and 12Sbi, [43]). The screening against the universal primer pairs was done to ensure that DNA could be successfully detected in all specimens. Amplifications were performed in total volumes of 25 µl, composed of 20.25 µl of 1× buffer (0.25 mM of each dNTP and 1.5 mM of MgCl_2_), 2.5 µl of primer mix (20 µM), 0.25 µl of *Taq* (i.e. 1.75 units) (Promega), and 2 µl DNA sample. The thermocycling program consisted of an initial step of 30s at 94°C (for *H. axyridis*, we used a hot start, i.e. addition of the *Taq* after the first step), followed by 30 s at 94°C, 30 s at 52°C (*H. axyridis*  =  55°C), and 30 s at 72°C. The three last steps were repeated 30 times and were followed by a step of 5 min at 72°C. All PCR products (10 µL) were electrophoresed at 120V in 2% agarose gels for approximately 1 h and then stained in ethidium bromide solution for 20 min and then visualized using a UV light-transilluminator. DNA is detectable at very low concentrations (from 35.5 ng × 10^−4^ to 35.5 ng × 10^−6^ depending on species primers) under optimal conditions (without heterospecific DNA).

Prey DNA detection success over time (DS_50_, the time after which 50% of the predators of a cohort that fed at the same time test positive for the presence of a species of prey using the PCR assay) ranged between 5.2 h and 19.3 h among combinations of interacting coccinellid species [30]. For this reason, corrected data using DS_50_ values for each predator-prey combination need to be used when comparing intensity of IGP between different coccinellid species. Such a correction confers more importance to a “rapidly digesting” species combination where probability of detecting an intraguild prey is lower than for a “slowly digesting:” species combination [44]–[46]. DS_50_ values for each predator-prey combination were weighted to obtain the DS_50_^weighted^ as follows: the shortest DS_50_ was assigned a value of 1.0 and other weighted DS_50_ values were obtained by placing this benchmark DS_50_ in the numerator and each other DS_50_ value in the denominator. The corrected predation value is calculated by multiplying the proportion of field-collected predators found to contain prey remains by their specific DS_50_^weighted^. We did not attempt to estimate amount eaten per predator because no strong relationship had been found between the number of prey eaten and the duration of DNA in gut-contents of coccinellids [29], [41].

## Results

**Figure 2:**
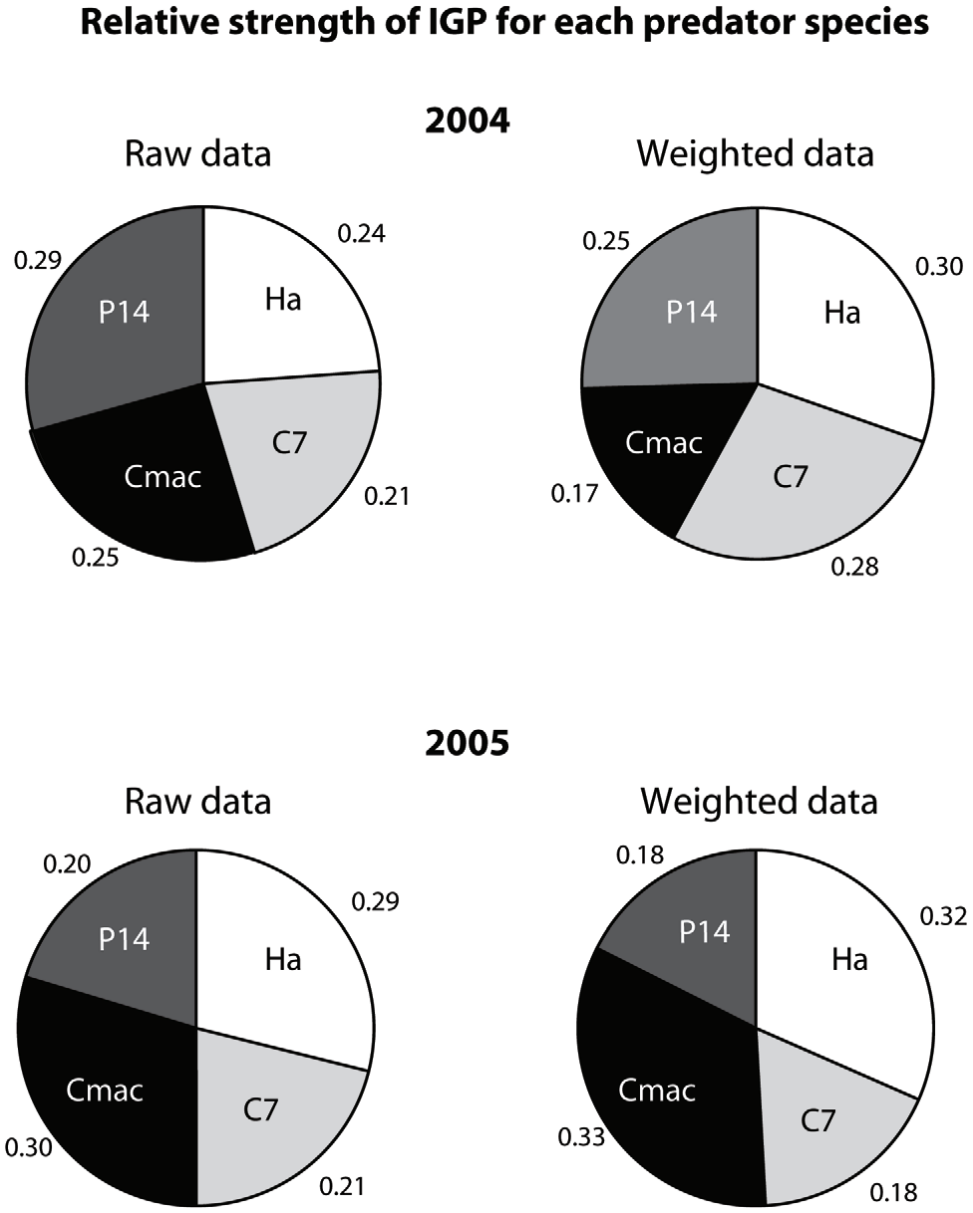
Relative strength of intraguild predation by each of the four coccinellid species measured by molecular gut content analysis in soybean fields in Québec, Canada, in 2004 and 2005. Results are shown for raw and weighted data*. Ha =  *Harmonia axyridis*, C7* =  Coccinella septempunctata*, Cmac* =  Coleomegilla maculata*, P14 =  *Propylea quatuordecimpunctata*.

Three novel results emerge from our study. First, levels of IGP were extremely high with averages of 46.8% and 58.9% (non-weighted data) of all coccinellids containing DNA of other coccinellids in their gut in 2004 and 2005, respectively (Table 1). The intensity of IGP for each coccinellid-coccinellid interaction, expressed as the proportion of each species of IG prey detected in the gut of IG predators is shown in Figure 1. Using the weighted DS_50_ values changed the ranking of predators in terms of IGP strength quite drastically in 2004 but only slightly in 2005. In 2004, the ranking using raw data was as follows: *P. quatuordecimpunctata* > *C. maculata* > *H. axyridis* > *C. septempunctata* (Figure 2). Using weighted DS_50_ values revealed the following ranking: *H. axyridis* > C. *septempunctata* > *P. quatuordecimpunctata* > *C. maculata* (Figure 2). Thus, using raw data leads to an underestimation of the relative importance of IGP by *H. axyridis* and *C. septempunctata*. In 2005, relative IGP rates were more similar among species. The ranking using raw was: *C. maculata* > *H. axyridis* > *C. septempunctata* > *P. quatuordecimpunctata* (Figure 2), which was almost unaltered when weighted DS_50_ values were used, except that the relative strengths of IGP for *C. septempunctata* and *P. quatuordecimpunctata* were the same (Figure 2).

**Table 1:** Number (N) of specimens tested and levels of intraguild predation (raw data) among four coccinellid species with molecular gut-content detection of one to three different intraguild prey species in a same predator, in 2004 and 2005.

	N	One intraguild prey species		Two intraguild prey species		Three intraguild prey species		Total IGP	
		*n*	%	*n*	%	*n*	%	*n*	%
2004	188	72	38.30	14	7.45	2	1.06	88	46.81
2005	180	74	41.11	29	16.11	3	1.67	106	58.89

Second, the results indicate that IGP is reciprocal with each of the four coccinellid species feeding on each of the other three species (Figure 1). However, although levels of IGP were high in both years, the relative proportion of intraguild prey species varied between years. In 2004, *H. axyridis* was strongly represented as an intraguild prey species, whereas in 2005 *P. quatuordecimpunctata* and *C. septempunctata* were the dominant intraguild prey species.

Third, we report multiple prey detection (Table 1). When results from both years are combined, 11.8% of the intraguild predators contained the DNA of two other coccinellid species in their gut, and we detected three intraguild prey species simultaneously in the guts of 1.4% of the sampled coccinellids. Consumption of two intraguild prey species was most common in *H. axyridis* (48.1% of all cases) and *C. maculata* (35.7%), whereas only *H. axyridis* was feeding on three intraguild prey species.

## Discussion

Our results indicate that IGP is very common among coccinellid species in soybean fields. Levels of IGP were high, with 52.9% of all sampled individuals containing the DNA of one, two and even three other coccinellid species in their gut. The interaction is reciprocal, as each of the four coccinellid species has the capacity to feed on the other three species. To our knowledge, this study represents the most convincing field evidence of the prevalence of IGP among predatory arthropods.

Our demonstration reflects the reality of the field situation. We used a sampling technique that entails no perturbation to the ecosystem or to the members of the community. Coccinellids were sampled *in situ*, without altering their behavior or distribution**,** thereby reducing potential artifacts that invariably arise through experimental manipulations conducted under laboratory conditions or within field cages. Molecular analyses allow the detection of minute amounts of prey material by PCR after DNA extraction. Molecular gut-contents analyses led to a demonstration of complex predation events between co-existing species and open the opportunity to better understand the dynamics and structure of communities. However, molecular gut-content analyses have their limits as well [23]. First, it is very difficult or impossible to determine the number of prey items a given predator has consumed, even using quantitative PCR [37], [47]. This is because the size of prey items and the degree of digestion per prey item can vary so widely. For this reason, the ecological significance of intraguild predation can be difficult to determine because we cannot compare the amount of intraguild prey eaten in relation to the extraguild prey. However, using DS_50_ correction allowed a comparison of intraguild predation rates between predator species that have different digestion times [30], [45], [46]. Second, scavenging or secondary predation (in which a predator eats another predator species containing the prey of interest in its gut) cannot be discriminated from true predation using PCR [48], [49]. And lastly, PCR detection of cannibalism is not achievable because conspecific DNA cannot be discriminated from predator DNA. Thus, we still lack a basic understanding of the relative importance of IGP and cannibalism, a common phenomenon in Coccinellidae [50], [51] for population dynamics. Monoclonal antibody-based ELISA could be useful in detecting cannibalism because it can be used to distinguish different life stages [52], [53].

While IGP models of predator-predator interactions, as well as the effects of omnivory on extraguild prey suppression have recently received considerable attention from both empiricists and theoreticians [2], [6], [7], [19], [54]-[57] very few studies have explicitly measured levels of IGP in arthropods under field conditions. To our knowledge only three other field studies using molecular techniques have directly quantified levels of IGP in arthropods. In the soybean agroecosystem, Harwood *et al.* [58] examined predation between *H. axyridis* and the predatory bug *Orius insidiosus* (Say) (Hemiptera: Anthocoridae) using molecular gut-content analysis. Less than 2.5% of *O. insidiosus* tested positive for the detection of *H. axyridis*. Chacon *et al.* [17] detected aphid parasitoid DNA in two predator species using PCR in a study examining IGP of released parasitoids of the soybean aphid. In this study, percentages of predators testing positive for parasitoid DNA ranged from 8 to 17. Hautier *et al.* [59] reported that 9 out of 28 *H. axyridis* collected in potato fields had fed on heterospecific species of coccinellids, based on alkaloid quantification by gas-chromatograph-mass spectroscopy (GC-MS). Although this latter technique is promising, identification of prey species is only possible at the genus level and this method has also been estimated to be more expensive than other analyses of gut contents [60]. More information about IGP levels measured under natural conditions is available for larger predators from different taxa (see Table 2 for selected examples), probably because predation events can be more easily detected through different sampling techniques. The first published study quantifying the incidence of IGP in nature was conducted by Polis and McCormick [4] who observed relatively high proportions of intraguild prey in the diet of desert scorpions, from 8 to 21.9%, and up to 45% for the species *Paruroctonus mesaensis.* Feeding information was easily collected on scorpions through observation because they digest their prey externally. Nevertheless, available data, both for arthropods and other taxa containing predators, are still too sparse to suggest patterns about the relative strength of IGP.

**Table 2:** Selected examples of intraguild predation under field conditions among different taxa.

IG predator	IG prey	Extraguild prey	% IGP	Method of detection	Region	Authors
White-tailed sea eagle (*Haliaeetus albicilla* L.)	Mink (*Mustela vison* Schreb.)	Fish and birds	<7% (for all mammal species)	Behavioral observation	Finland	[62], [63]
Cougar, wolf	Coyote	Small mammals	43–67%	Radio-tracked animals	Alaska, Idaho	[64]
Lion, spotted hyena	African wild dog		13–50%		South Africa, Tanzania	
Red fox	American marten		4%		Ontario	
Scorpion *Paruroctonus mesaensis*	*P. luteolus* *H. arizonensis* *V. confuses*	Insects	8–22% (in some months higher than 40%)	External digestion (direct observation)		[4]
Eagle owl	Tawny owl	Mammals, birds, fish, invertebrates	0.6%	Pellets and prey remains found under nests and roost sites	Italy	[65]
Dingo	Feral catRed fox	NA	1.2–6.1%	Dissection of gut-content	Australia	[66]
Many intertidal herbivores	Many intertidal herbivores	NA	0.37–10%	Dissection of intestinal content	Chile	[67]

Several factors may contribute to the very high levels of IGP we quantified in coccinellids. First, coccinellids respond numerically to high aphid densities [24]-[27] a condition that may favour encounters between predators; although high prey abundance may also lead to predator satiation and thereby a reduction in intraguild interactions. Second, by eating a heterospecific, intraguild predators eliminate a competitor and thereby improve access to the aphid resource. Third, aphids are a relatively low quality prey resource [61], and coccinellids may benefit by complementing their diet by feeding on other coccinellids. A recent study also showed high levels of predation on coccinellid eggs in soybean fields in Michigan, USA [18]. However we still have a poor understanding of ecological factors that influence the strength and direction of intraguild interactions, and there is a need for more empirical studies that examine the effect of factors such as seasonality, vegetation-structured complexity, habitat productivity, extraguild prey density, as well as the behaviors and life histories of protagonists.

Over the last 20 years, several models and experimental studies have examined the nature and role of intraguild interactions in both terrestrial and aquatic communities. Intraguild predation is now considered to be ubiquitous in most species assemblages [12]. However, previous studies conducted in natural or managed ecosystems have largely overlooked the prevalence of IGP among top predators. Our results on coccinellids emphasize the importance of quantifying IGP in the field. This basic information is central for understanding the role of top predators in population dynamics and community structure, and from a more applied perspective, to predict their impact in programs devoted to the biological control of pest species or the management of native endangered or invasive exotic species.
